# Factors underlying the polarization of early childhood caries within a high-risk population

**DOI:** 10.1186/1471-2458-14-988

**Published:** 2014-09-22

**Authors:** Ana Margarida Melo Nunes, Antônio Augusto Moura da Silva, Claudia Maria Coelho Alves, Fernando Neves Hugo, Cecilia Claudia Costa Ribeiro

**Affiliations:** Federal University of Maranhão, Jupiter Street 12, apartment 1101, Ed. José Gonçalo, Renascença II, São Luis, Ma Brasil; Federal University of Rio Grande do Sul, Porto Alegre, Brazil

**Keywords:** Early childhood caries, Public dental health, Diet, Epidemiology

## Abstract

**Background:**

Early childhood caries (ECC) are particularly prevalent in disadvantaged populations, and socioeconomic factors are associated with the polarization of disease. A previous study showed that even within a homogenous low-income population disease is polarized, indicating that other factors apart from income may contribute to disease susceptibility.

**Methods:**

This study used a hierarchical approach to identify factors associated with polarization of ECC in low-income subjects. This cross-sectional study was conducted retrospectively using a cohort of 244 children (aged 48–72 months) with family incomes not exceeding double the minimum wage (U.S. $8,208.00/year), living in neighborhoods on the outskirts of São Luís, Maranhão, Brazil. The sample was divided into three groups based on the Significant Caries (SiC) Index: no caries group, few caries group (mean 1.38 lesions), and a high caries group (mean 3.82 lesions). Hierarchical multinomial logistic regression analyses were performed based on a theoretical model.

**Results:**

Twenty-eight (11.5%) of the 244 children presented with high caries. Age (*p* = 0.026; prevalence ratio (PR) = 1.10; 95% confidence interval (CI) 1.01–1.20) and frequency of sucrose consumption - *p* = 0.001; PR 4.65 (95% CI 1.83–11.84) were associated with increased risk of ECC.

**Conclusions:**

In the high caries group, greater consumption of sucrose between main meals may explain why, in a group of children with homogenous social and health conditions, some had more caries than others.

## Background

Early childhood caries (ECC) is a disease that involves development of one or more caries lesions, with or without cavitation, by the age of 71 months [1]. Epidemiological studies have indicated that ECC has a strong social element [2–4]. Indeed, higher prevalence in non-developed countries [5, 6] and in socioeconomically disadvantaged groups living in low, middle [2–4] and high-income countries [7, 8] have been documented.

Increased concentration of ECC within a specific group signifies a polarizing phenomenon, defined as a greater number of cases within a small segment of the population [9–11]. Polarization of caries has often been associated with socioeconomic deprivation in populations at social risk [9, 11–13]. This phenomenon may result from the non-homogenous distribution of strategies to control caries in populations, such as improvement in socioeconomic status and access to preventive dental care, fluoride, and dental education programs [9].

Polarization of ECC is well documented; the majority of young children are caries-free, and most carious lesions are concentrated in a small number of children [14–16]. The concentration of caries disease in a segment of the population has also been demonstrated in Brazil, in preschool [11] and school age children [9, 17]. The phenomenon is also associated with socioeconomic determinants in this population. In addition, polarization of caries disease has also been documented among Brazilian adolescents [10, 18] associated with skin color and the presence of tooth pain in the previous half year [10].

In a previous study involving children 18 to 42 months of age [19], we observed that caries disease was polarized in a population characterized by low socioeconomic status in the peripheral neighborhoods of São Luís, Maranhão Brazil. Given that socioeconomic factors have been associated with ECC risk and its polarization among children, it is important to understand what factors mediate susceptibility to the disease. Elucidation of these factors may reveal why, within a group of children who are all vulnerable, some have more caries than others.

Identifying modifiable proximal factors that mediate the association between socioeconomic status (SES) and ECC polarization may help design interventions to ameliorate the burden of this disease, especially among low-income groups. To the best of our knowledge, no previous study has explored proximal factors that may mediate the association between SES and caries polarization. The objective of this study was to use a hierarchical approach to identify factors associated with ECC polarization within a vulnerable low-income population.

## Methods

### Study design

This was a cross-sectional study nested within a cohort study of preschool children aged from 18 to 42-months, in which was evidenced caries polarization [19]. This study was conducted from March to September 2008 in the city of São Luís, Maranhão, Brazil. A convenience sample of children enrolled from community nurseries servicing low-income families, with incomes up to twice the Brazilian minimum wage (corresponding to U.S. $8,208.00/year) was selected. Four nurseries located in the poorest city district were sufficient to complete the sample size. All children attending these nurseries were invited to participate in the study.

A total of 260 parents signed informed consent forms for participation in the study. Exclusion criteria included the presence of debilitating systemic diseases, malnutrition, and antibiotic use that could interfere with colonization of *Streptococcus mutans* (SM) 30 days prior to the onset of the study. These factors were chosen because of their potential to influence the expression of caries.

Considering that this study used previously collected data, [19] ad hoc power analysis indicated that a sample of 226 children is able to detect a 2.5 prevalence ratio (PR), with an 80% test power and a 5% probability of type I error, assuming that 10% of children presented caries.

### Data collection

The mothers or caregivers were interviewed using a structured questionnaire that was tested in a pilot study involving 25 mothers or caregivers. The questionnaire included demographic and social variables (mother’s age, mother’s years of education, family income), mother-child data (if the mother has untreated cavities of caries or not), who stays at home with the child, previous information about oral health (if she received information about oral health care by a dentist or by health professional), previous child hospitalization, antibiotic use in the last year by the child, child visits to the dentist, nighttime breastfeeding after 1 year and child oral hygiene practices (when teeth brushing started, who brushes the child’s teeth and daily toothbrush frequency).

A 24-hour food recall was used to acquire information about participants’ dietary habits (use of a night bottle and frequency of sucrose consumption between main meals in the form of juice, baby formula, snacks, and sweets). On a separate day, a dental surgeon assessed the visible plaque index (VPI) of each participant under natural light in the knee-to-knee position. To calculate VPI, a score corresponding to the sum of tooth sides with clinically visible plaque in relation to the total number of tooth sides present was determined [20].

The same examiner determined the number of teeth with carious decay, with extraction completed or indicated, and with fillings apparent by surface examination (dmfs index) for each child using a dental mirror (n° 5) and an exploratory probe with a rhombus point, as recommended by the World Health Organization [21]. The clinical exam was repeated one week later in 10% of the study population to evaluate intra-examiner agreement (kappa = 0.91). To identify the group of children in which caries disease was polarized, the Significant Caries (SiC) Index, adapted for deciduous teeth corresponding to the third of the sample with highest dmfs scores, was used [22].

To detect SM contamination, 1.7 mL of non-stimulated saliva was collected by pressing a sterile swab on the sublingual zone and dorsal tongue of each child until the swab was saturated (~5 seconds in each area). Each saliva sample was diluted in saline solution (0.9% NaCl) to produce decimal dilutions. Aliquots (0.1 mL) of each dilution were spread onto Mitis salivarius-bacitracin agar plates and incubated in a 5% CO_2_ chamber for 48 hours. After incubation, the number of colony-forming units (CFUs) was counted on each plate with >10 colonies. A single examiner, who did not have knowledge of patients’ caries status, performed the microbial counts in duplicate using a digital colony counter (model CP 600 Plus, Phoenix).

### Theoretical model

**Figure 1:**
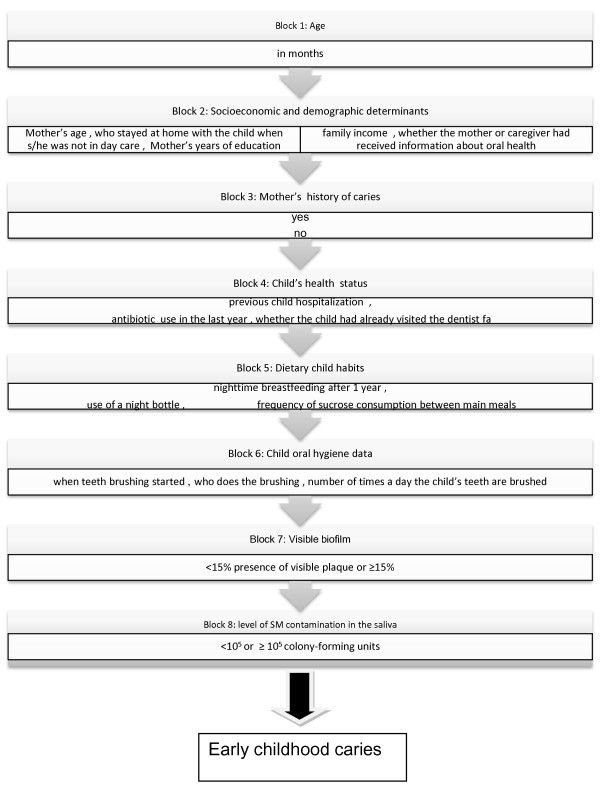
**Hierarchical theoretical model of factors associated with polarization of early childhood caries.**

A hierarchical theoretical model with the following eight blocks was employed: 1) age of child in months; 2) socioeconomic and demographic factors; 3) mother’s past history of caries (if the mother has untreated cavities of caries or not); 4) child’s health status; 5) dietary child habits; 6) child oral hygiene data; 7) VPI; and 8) level of SM contamination in the saliva (Figure 1).

Age was considered a potentially confounding factor, so the model was adjusted for this variable, which represented the block furthest from the outcome. This variable was added as a continuous variable.

The second block included socioeconomic and demographic variables as distal factors in the theoretical model since they can influence all variables in subsequent blocks. Were included in this block mother’s age in years, mother’s years of education (<8, 8–11, or >11), family income (<1 minimum wage, 1 minimum wage (U.S. $4,104.00/year), or >1 minimum wage), who stayed at home with the child when she/he was not in day care (the mother, others), and whether the mother or caregiver received information about oral health (yes or no) [19].

The health variable included for the mother in the third block, which may mediate the association between socioeconomic variables and childhood caries, was previous history of caries, self-reported by the mother. She reported if she had untreated cavities of caries or not at the time of interview (yes, no).

Health variables for the child were included in the fourth block since they may mediate the association between the variables included in the previous blocks and childhood caries. The following variables were used: previous child hospitalization reported by the mother or caregiver (0, 1, or ≥2 times), antibiotic use in the last year (yes, no), and whether the child had already visited the dentist (yes, no).

In the fifth block, dietary practices were included, assuming that these variables may be influenced by socioeconomic and demographic factors, and by mother and child health variables. The variables in this block were: nighttime breastfeeding after 1 year (yes, no), use of a night bottle (yes, no), and frequency of sucrose consumption between main meals in the form of juice, baby formula, snacks, and sweets (≤3 or >3 times/day).

Oral hygiene practices may be moderator variables of the association between diet and ECC, so they were included in the sixth block as follows: when teeth brushing started (0–6 months, 7–12 months, after 12 months), who does the brushing (the child alone, the caregiver), and the number of times a day the child’s teeth are brushed (up to 1 time, ≥2 times).

In the seventh block, whether a visible biofilm was present (<15% presence of visible plaque or ≥15%) was recorded assuming that this variable may be influenced by both hygiene and dietary practices.

In the final block the level of SM contamination in the saliva (<10^5^ or ≥10^5^ CFU – colony-forming units) was included as a biological variable nearest to the outcome that might be influenced by all variables in the previous blocks.

### Statistical analyses

Multinomial logistic regression using forward selection and guided by a hierarchical approach was used [19, 23]. We evaluated possible associations between risk factors and ECC using a series of models, which allowed for the modeling of the dependent variable, structured as polytomous data divided according to three SiC categories (caries-free, few caries, high caries). The estimated coefficients were expressed as prevalence ratios (PRs) and their 95% confidence intervals were also calculated.

The hierarchical modeling started with the first block. The variables of the first block were adjusted simultaneously for each other and only those variables whose p value was <0.10 entered in subsequent models. Then variables of the second block were adjusted simultaneously for each other and for the variables whose p value was <0.10 in the previous step. The significance of each variable was considered at the time of entry into the model (p value <0.05). All other blocks were then added in succession following the same procedure.

All statistical analyses were performed using the STATA 10.0 program (Stata Corp., College Station, TX). Collinear variables were evaluated using the variance inflation factor.

### Ethical aspects

The ethics committee of the University Hospital of the Federal University of Maranhão (Universidade Federal do Maranhão, UFMA) (case 33104-1251/2007) approved the study. The parents or legal representatives of each child that participated in the study read and signed informed consent forms. All children needing dental treatment were referred to the pediatric dental clinical of the UFMA.

## Results

Among the 260 children whose parents signed informed consent forms, 16 (6.2%) were excluded from the study because they did not complete all steps of the investigation. In our final analyses, we used data from 244 children. In this study, ECC prevalence was 32%. Mean dmfs index was 0.42 and the standard deviation (SD) was 0.69 in the whole sample. Separating the dmfs index into their components, only the component cavity was observed in our sample.

According to the SiC index, the high caries group (more than three caries lesions) included 28 children (mean 3.82 lesions, SD 0.45). The few lesions group (1-3 caries lesions) contained 49 children (mean 1.38 lesions, SD 0.49). A distribution of explanatory variables according to caries group is shown in Table 1.

**Table 1:** **Distribution of variables in the three groups: caries free, few lesions and high caries group**

Variables	Caries free group N (%)	Few lesions group N (%)	High caries group N (%)
Family Income (minimum wages)			
<1	55 (65)	19 (23)	10 (12)
1	75 (68)	22 (20)	14 (13)
> 1	30 (79)	5 (13)	3 (8)
Mother’s years of education			
< 8	37 (59)	16 (25)	10 (16)
8 a 11	64 (72)	15 (17)	10 (11)
> 11	59 (73)	15 (18)	7 (91)
Who stayed at home with the child when s/he was not in day care			
The mother	80 (68)	24 (20)	13 (11)
Others	80 (69)	22 (19)	14 (12)
Whether the mother or caregiver had received information about oral health			
No	68 (71)	18 (19)	9 (9)
Yes	93 (67)	28 (20)	18 (137)
Mother’s history of caries			
No	68 (69)	21 (22)	9 (9)
Yes	92 (68)	25 (19)	18 (13)
Number of previous child hospitalizations			
0	90 (67)	27 (20)	18 (13)
1	55 (71)	15 (20)	7 (9)
≥2 times	16 (73)	4 (18)	2 (9)
Whether they had already visited the dentist			
No	135 (67)	44 (22)	22 (11)
Yes	26 (79)	2 (6)	5 (15)
Antibiotic use in the last year			
No	107 (70)	26 (17)	19 (13)
Yes	54 (66)	20 (24)	8 (10)
Nighttime breastfeeding after 1 year			
No	125 (66)	45 (24)	20 (10)
Yes	38 (78)	4 (8)	7 (14)
Use of a night bottle			
No	117 (65)	40 (22)	23 (13)
Yes	44 (82)	6 (11)	4 (7)
Frequency of sucrose consumption between main meals			
≤ 3	145 (72)	42 (21)	15 (7)
>3 times	22 (55)	6 (15)	12 (30)
When teeth brushing started (months)			
0 - 6	19 (86)	3 (14)	0 (0)
7 a 12	69 (69)	16 (16)	15 (15)
> 12	73 (65)	27 (24)	12 (11)
Who does the teeth brushing			
The child alone	17 (49)	14 (40)	4 (11)
The caregiver	144 (72)	32 (16)	23 (11)
The number of times a day their teeth are brushed			
up to 1	15 (58)	7 (27)	4 (15)
≥2	146 (70)	39 (19)	23 (11)
Visible biofilm			
<15%	58 (79)	11 (15)	4 (6)
≥ 15%	112 (64)	38 (22)	24 (14)
Streptococcus mutans contamination in the saliva			
<10^5^ CFU*	104 (69)	28 (19)	17 (11)
≥ 10 ^5^ CFU*	63 (66)	21 (22)	11 (12)
Total**	167 (100)	49 (100)	28 (100)

*CFU: colony-forming units **umbers may not add to total because of missing values.

The results of the multinomial model of hierarchized regression are reported in Table 2. No collinear variables were identified. In block 1, the age of the child was significantly associated with ECC in the high caries group (*p* = 0.026; PR = 1.10; 95% CI 1.01–1.20) and this association in few caries group was found to be within the limit of significance (*p* = 0.054; PR = 1.06; 95% CI 1.00-1.12). In the second and third blocks, none of the variables were associated with the outcome. In the fourth block, having access to a dentist was an indicator of protection in the few caries group (*p* = 0.035; PR = 0.20; 95% CI 0.05–0.89) but not in the high caries group (*p* = 0.932). In the fifth block, nocturnal breastfeeding beyond the age of 1 year was in the limit of significance, as a borderline protective factor for the few caries group (*p* = 0.051; PR = 0.33; 95% CI 0.11–1.01). In this same block, the consumption of sucrose between main meals was significant (*p* = 0.001) in the high caries group, PR = 4.65 (95% CI 1.83–11.84) (Table 2).

**Table 2:** **Multinomial logistic regression of the factors associated with early childhood caries (group with few lesions and high caries group) in a hierarchical model**

Groups/Variables	Block 1		Block 4		Block 5		Block 6	
	PR (95% CI)	p	PR (95% CI)	p	PR (95% CI)	p	PR (95% CI)	p
**Few caries group**								
Age	1.06 (1.00-1.12)	0.054						
Child has visited the dentist								
No			1					
Yes			0.20 (0.05-0.89)	0.035				
Frequency of sucrose consumption between main meals								
≤3					1			
>3					0.83 (0.28-2.42)	0.732		
Use of a night bottle								
No					1			
Yes					0.42 (0.16-1.08)	0.073		
Nighttime breastfeeding after 1 year								
No					1			
Yes					0.33 (0.11-1.01)	0.051		
Who does the teeth brushing								
The child alone							1	
The caregivers							0.27 (0.11-0.66)	0.004
**High caries group**								
Age	1.10 (1.01-1.20)	0.026						
Child has visited the dentist								
No			1					
Yes			1.05 (0.36-3.09)	0.932				
Frequency of sucrose consumption between main meals								
≤3					1			
>3					4.65 (1.83-11.84)	0.001		
Use of a night bottle								
No					1			
Yes					0.57 (0.18-1.85)	0.353		
Nighttime breastfeeding after 1 year								
No					1			
Yes					1.38 (0.50-3.80)	0.532		
Who does the teeth brushing								
The child alone							1	
The caregivers							1.00 (0.25-4.02)	0.998

*PR* prevalence ratio, *CI* confidence interval.

Only variables whose p value were < 0.10 were shown in the table and others described below were omitted. Results for each variable were shown only for the block at which the variable was entered first. Blocks 2, 3, 7 and 8 were not presented because no significant variables were kept at these blocks.

Block 1: adjusted for age.

Block 2: adjusted for variable kept in block 1(age) plus variables from block 2.

Block 3: adjusted for variable kept in block 1(age) plus variables from block 3.

Block 4: adjusted for variable kept in block 1(age) plus variables from block 4.

Block 5: adjusted for variable kept in block 1(age), variable selected from block 4 (child has visited the dentist) plus variables from block 5.

Block 6: adjusted for variable kept in block 1(age), variable selected from block 4 (child has visited the dentist), variables selected from block 5 (frequency of sucrose consumption between main meals, nighttime breastfeeding after 1 year, and use of a night bottle) plus variables from block 6.

Block 7: adjusted for variable kept in block 1(age), variable selected from block 4 (child has visited the dentist), variables selected from block 5 (frequency of sucrose consumption between main meals, nighttime breastfeeding after 1 year and use of a night bottle), variable selected from block 6 (who does the teeth brushing) plus variables from block 7.

Block 8: adjusted for variable kept in block 1(age), variable selected from block 4 (child has visited the dentist), variables selected from block 5 (frequency of sucrose consumption between main meals, nighttime breastfeeding after 1 year and use of a night bottle), variable selected from block 6 (who does the teeth brushing) plus variables from block 8.

In the sixth block, the variable of the mother or caregiver brushing the child’s teeth for them was a significant protective factor in the few caries group (*p* = 0.004; PR = 0.27; 95% CI 0.11–0.66) but was not significant in the high caries group. The biological variables in the seventh (VPI) and eighth blocks (*S. mutans* contaminated saliva) were not associated with childhood caries.

## Discussion

This study revealed that high sucrose consumption was associated with caries polarization among children living in vulnerable social condition after controlling for confounding variables through a hierarchical approach.

The phenomenon of caries polarization among children has been associated with social deprivation [24]. However it has been shown that even in populations with homogenous social background caries tend to concentrate in certain population subgroups [9, 24]. Reasons for this are poorly understood. In the present study we showed that higher sugar consumption is possibly the factor that mediates the association between low SES and caries polarization.

Indeed, studies have indicated that low socioeconomic level can alter dietary patterns [25] so sugar consumption may be a pathway to the association between low family income and caries polarization, since both socioeconomic factors [3, 4] and sucrose consumption [4, 15, 18, 19, 25, 26] also present robust associations with ECC. It is possible that synergy between deprived social conditions and high-sugar dietary patterns may explain these findings.

In the present study, we used the SiC index to identify the high caries group as a proxy for disease polarization, showing a mean of 3.82 lesions. Other studies involving children up to five years of age showed a SiC value similar to ours, such as a SiC value of 4.14 in Mexican children [27] and of 4.09 in Sri Lankan children [12].

Ours results showed that age was associated with ECC, in the high caries groups, confirming results of previous studies indicating that caries severity increases with age [19, 28–32].

The demographic, economic, and social variables (income, schooling, health information) in the second level were not associated with ECC. These data differ from results of two prior studies [2, 3], which found that social variables were associated with ECC. This is likely explained by the homogeneity of social variables in the cohort included in this study. Although the homogeneity lessened the power of social variables, homogeneous social variables are desirable in a study investigating variables that explain polarization of ECC incidence to reduce confounding by SES.

In the few caries group, the non-polarization phenomenon may be attributable to several protective factors, including access to a dentist, being breastfed after the age of 1 year and teeth brushing by the mother. Thus, it seems that even in deprived low-income groups, access to health care, protective feeding practices and oral hygiene might buffer the deleterious effect of high sugar consumption on ECC.

Access to the dentist was protective factor only in the few caries group (PR = 0.20). It’s possible that children of high caries group may have gone to the dentist for restorative care or even due to toothache. In this sense, it has been shown that access to dental treatment among preschoolers occurs especially for emergency care [33].

Similarly, teeth brushing performed by the mother were also protective only for the few caries group (PR = 0.27). The high caries group may not have had adequate hygiene practices, resulting in higher DMFS scores.

Nocturnal breastfeeding after age 1 (PR = 0.33) was a protective factor of borderline significance in the group with few caries (p = 0.051). It is possible that the small sample size could have contributed to the borderline association between nocturnal breastfeeding and ECC observed in the group with few caries. In the high caries group, however, this practice was not found to protect from the disease, possibly because children with greater number of lesions had other deleterious dietary practices superimposed on breastfeeding, such as consumption of more sucrose. Others epidemiological studies failed to show that breastfeeding after age 1 were associated with ECC [34–36], including previous analysis of data involving the same population of children [19].

The fact that we used a convenience sample is a limitation to the external validity of this study’s findings. However, it is possible that our results could be extrapolated to other low SES populations living in similar conditions.

One of the strengths of our study is that since ECC is considered a social disease, studying a population with homogenous social and economic conditions favors the identification of factors that stratify risk within vulnerable groups and allows for better control of confounding factors. Furthermore, the use of a hierarchical model permitted us to evaluate mediation of more proximal factors on the association between distal variables such as SES and ECC.

## Conclusion

In conclusion, higher sucrose consumption increases the likelihood of ECC in socially vulnerable children. Caries population prevention strategies based on teeth brushing and visits to a dentist seems not to be enough to reduce inequalities in oral health because they do not seem to be reaching the most vulnerable groups among the vulnerable. Another possible explanation is that these preventive practices are not sufficient to counterbalance the deleterious effect of high sugar consumption in the high-caries group of children. Public policies focused on warning about the risk of added sugars consumption might be an additional strategy to control ECC in these deprived low-income populations.
